# Testicular Cancer–Associated Paraneoplastic Neurologic Syndromes

**DOI:** 10.1001/jamanetworkopen.2025.38584

**Published:** 2025-10-21

**Authors:** Ehab Harahsheh, M. Bakri Hammami, Pranjal Gupta, Brian A. Costello, Bradley Leibovich, John C. Cheville, Anastasia Zekeridou, Andrew McKeon, Sean J. Pittock, Divyanshu Dubey

**Affiliations:** 1 Division of Neuro-Oncology, Department of Neurology, Mayo Clinic Arizona, Scottsdale; 2 Laboratory Medicine and Pathology, Mayo Clinic College of Medicine, Rochester, Minnesota; 3 Division of Medical Oncology, Mayo Clinic, Rochester, Minnesota; 4 Department of Urology, Mayo Clinic, Rochester, Minnesota; 5 Center for Multiple Sclerosis and Autoimmune Neurology, Department of Neurology, Mayo Clinic College of Medicine, Rochester, Minnesota

## Abstract

This cohort study examines the oncological, neurological, and serological profiles of adult male patients with testicular germ cell tumors and paraneoplastic neurologic syndromes.

## Introduction

Testicular germ cell tumors (TGCTs) are common solid tumors among young male adults. Paraneoplastic neurologic syndromes (PNSs) have been reported in this patient population,^1^ with several neural antibody biomarkers identified, including Ma2, Kelch-like protein 11 (KLHL11), and leucine zipper 4 (LUZP4) antibodies.^2,3,4,5^ Timely diagnosis and treatment of PNS may mitigate substantial long-term neurological disabilities and improve cancer outcomes. This study aims to describe the oncological, neurological, and serological profiles of patients with TGCT and PNS.

## Methods

This retrospective cohort study included patients aged 18 years or older with histopathologically confirmed TGCT, or regressed TGCT, and PNS. Patients were evaluated and treated at Mayo Clinic from January 1, 1990, to March 30, 2023. For eligible patients with TGCT and PNS, serum and/or cerebrospinal fluid were tested for neural antibodies, including KLHL11-IgG, LUZP4-IgG, and Ma2-IgG.^3,4,5^ Patients’ records were reviewed for demographic, oncological, and neurological data whenever available. Further information regarding consent, reporting guidelines, and statistical methods is provided in the eAppendix in Supplement 1.

## Results

In total, 49 patients (median [IQR] age, 41.0 [32.5-47.5] years) were included (45 with definite PNS and 4 with probable PNS, as per PNS CARE score). Most TGCT cases were seminomas (33 patients [67%]), with the remainder (9 patients [18%]) being nonseminomatous germ cell tumor (NSGCT) or regressed TGCT (7 patients [14%]) (Table). Over a median (IQR) of 33.0 (6.5-64.0) months from TGCT diagnosis to final follow-up, 80% of patients achieved either cure (18 patients [37%]) or remission (21 patients [43%]), 6 patients (12%) experienced tumor relapses, and 9 patients (18%) died. No deaths were attributed to TGCTs; 6 patients died of PNS, 1 died of myocardial infarction, and the cause was unclear for 2 patients.

**Table.** Oncological and Neurological Characteristics of the Cohort

Characteristic	Patients, No. (%)			
	Total (N = 49)	Seminoma (n = 33)	NSGCT (n = 9)	Regressed TGCT (n = 7)
Age at the time of TGCT diagnosis, median (IQR), y	41.0 (32.5-47.5)	41.0 (32.5-55.0)	34.0 (27.0-43.0)	44.0 (40.0-47.0)
Tumor location				
Gonadal	36 (74)	22 (67)	7 (78)	7 (100)
Retroperitoneal	6 (12)	5 (15)	1 (11)	0
Mediastinal	7 (14)	6 (18)	1 (11)	0
Tumor stage				
Stage 1	20 (47)	15 (45)	5 (56)	NA
Stage 2	15 (36)	12 (37)	3 (33)	NA
Stage 3	7 (17)	6 (18)	1 (11)	NA
TGCT prognosis per International Germ Cell Cancer Collaborative Group				
Good	35 (90)	30 (97)	5 (63)	NA
Intermediate	2 (5)	1 (3)	1 (12)	NA
Poor	2 (5)	NA	2 (25)	NA
Treatment received for TGCT				
Surgery	47 (96)	32 (97)	8 (89)	7 (100)
Radiation	13 (27)	13 (39)	0	0
Chemotherapy	15 (31)	11 (33)	4 (44)	0
Combination	25 (51)	22 (67)	3 (33)	0
Treatment response for TGCT				
Cure	18 (37)	9 (27)	2 (22)	7 (100)
Remission	21 (43)	16 (49)	5 (56)	0
Relapse	6 (12)	4 (12)	2 (22)	0
Stable	2 (4)	2 (6)	0	0
Unknown	2 (4)	2 (6)	0	0
Neurological presentation				
Ataxia	31 (63)	21 (63.6)	3 (33)	7 (100)
Sensorineural hearing loss^a^	22 (45)	18 (54.5)	0	4 (57)
Tinnitus^b^	15 (31)	12 (36.3)	0	3 (43)
Seizures^c^	12 (24)	6 (18.1)	5 (56)	1 (14)
Sleep disturbances^d^	10 (20)	3 (9)	7 (78)	0 (0)
Neurological outcomes				
Improvement	8 (16)	6 (18)	1 (11)	1 (14)
Stabilization	21 (43)	16 (49)	4 (44)	1 (14)
Progression	20 (41)	11 (33)	4 (44)	5 (72)
Mortality	9 (18)	6 (18)	1 (11)	2 (29)
Time from diagnosis to final follow-up, median (IQR), mo	33.0 (6.5-64.0)	36.5 (6.75-64)	33.5 (21.0-91.8)	1 (1-5)
Time from diagnosis to relapse, median (IQR), mo	14.5 (11.0-31.5)	12.0 (8.0-25.5)	53.5 (34.2-75.8)	NA
Time from initial diagnosis to death, median (IQR), mo	9.0 (4.0-46.0)	6.0 (3.5-8.8)	NA	29.5

Abbreviations: NA, not applicable; NSGCT, nonseminomatous germ cell tumor; TGCT, testicular germ cell tumor.

^a^ *P* = .005, seminoma vs NSGCT.

^b^ *P* = .04, seminoma vs NSGCT.

^c^ *P* = .009, seminoma vs NSGCT.

^d^ *P* < .001, seminoma vs NSGCT.

Ninety-four percent of patients tested positive for neural autoantibodies. The most frequently identified antibody was KLHL11-IgG (32 cases, 20 alone and 12 coexisting with LUZP4-IgG), followed by Ma2-IgG (9 cases, with 1 case coexisting with LUZP4-IgG), and LUZP4 IgG alone (5 cases) (Figure). In patients with KLHL11-IgG and LUZP4-IgG, seminomas were present in 72% (23 patients) and 78% (14 patients), respectively. However, 78% of patients with Ma2-IgG (7 patients) had NSGCT. KLHL11-IgG and Ma2-IgG were associated with seminomas (odds ratio [OR], 8.06; 95% CI, 1.42-45.46; *P* = .02) and NSGCTs (OR, 54.25; 95% CI, 6.48-454.07; *P* < .001), respectively.

**Figure.**
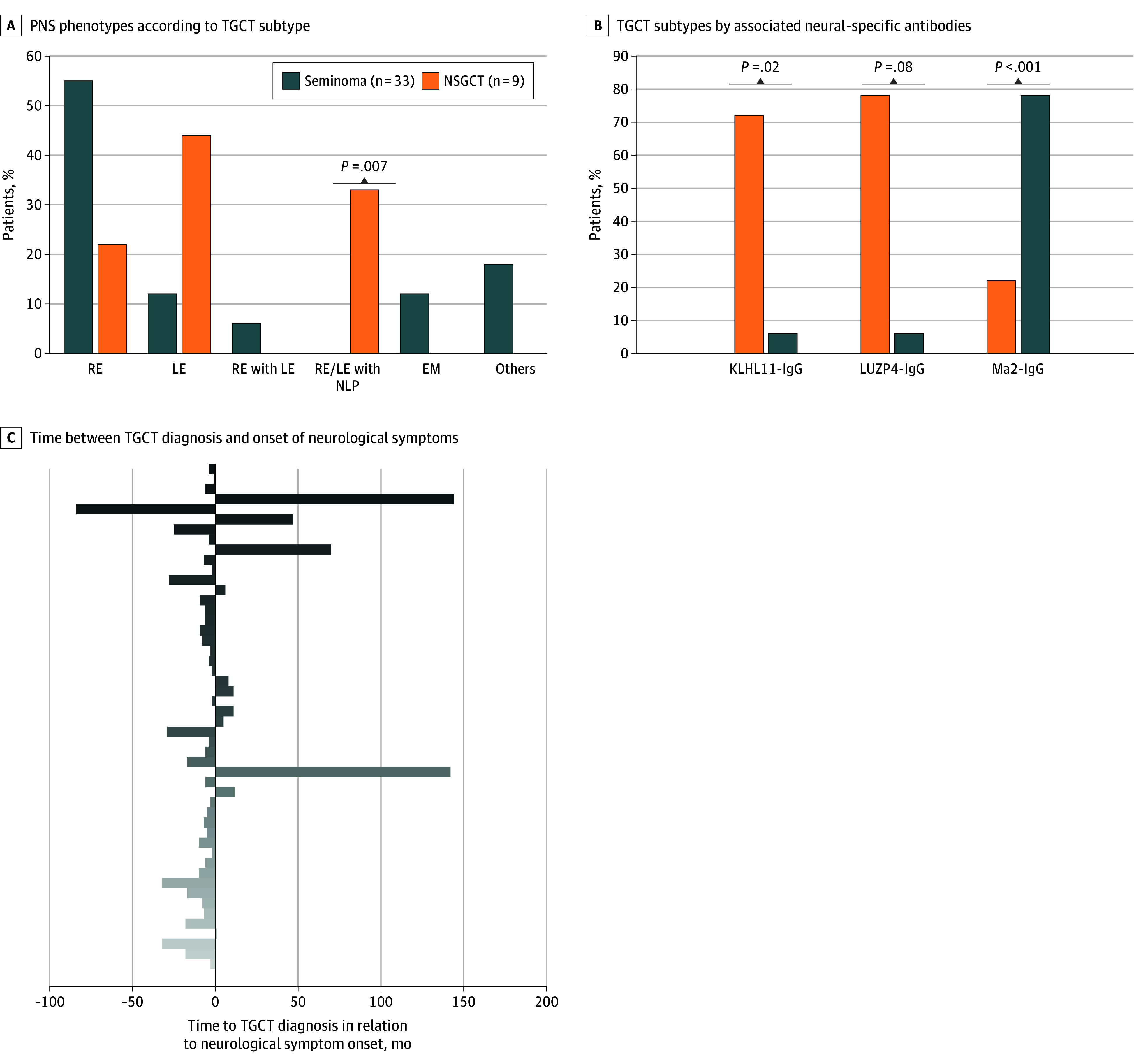
Phenotypic Patterns, Antibody Associations, and Temporal Relationship of Neurological Syndromes in Testicular Germ Cell Tumors (TGCTs) A, Paraneoplastic neurological syndrome (PNS) phenotypes, including rhomboencephalitis (RE), limbic encephalitis (LE), narcolepsy (NLP), and encephalomyelitis (EM), according to TGCT subtype (seminoma vs nonseminomatous germ cell tumors [NSGCTs]). Other phenotypes include autoimmune epilepsy (n = 1), motor neuron disease (n = 1), myeloradiculoneuropathy (n = 2), and peripheral neuropathy (n = 2). B, Distribution of TGCT subtypes by associated neural-specific antibodies, including Kelch-like protein 11 (KLHL11)–IgG, leucine zipper 4 (LUZP4)–IgG, and Ma2-IgG. C, Temporal relationship between TGCT diagnosis and the onset of neurological symptoms. Negative values indicate symptom onset before TGCT diagnosis; positive values indicate onset after diagnosis. Most patients (denoted by individual bars) developed neurological symptoms before TGCT was diagnosed.

Most patients (33 patients [80%]) experienced PNS symptoms before TGCT diagnosis (Figure). Ataxia (31 patients [63%]), diplopia (29 patients [59%]), sensorineural hearing loss (22 patients [45%]), and vertigo (20 patients [41%]) were the most observed symptoms. Seminomas frequently presented with ataxia, sensorineural hearing loss, and vertigo. Conversely, NSGCTs presented more frequently with seizures and sleep disturbances (Figure). KLHL11-IgG was associated with sensorineural hearing loss (OR, 12.50; 95% CI, 2.43-64.43; *P* < .001) and tinnitus (OR, 12.44; 95% CI, 1.47-105.52; *P* = .006), whereas Ma2-IgG was associated with seizures (OR, 11.33; 95% CI, 2.21-58.15; *P* = .004) and sleep disturbances (OR, 43.17; 95% CI, 6.06-307.41; *P* < .001). Only 8 patients (16%) showed improvement of their PNS, while 21 (43%) had stabilization and 20 (41%) had progression, despite immunomodulatory treatment.

## Discussion

This cohort study found that 80% of patients had neurological symptoms before TGCT diagnosis. Thus, recognition of early signs and symptoms of PNS in young and middle-aged patients should alert neurologists not only to test for KLHL11-IgG, LUZP4-IgG, and Ma2-IgG, but also to search for any underlying occult malignant entity, especially TGCTs. Early recognition and diagnosis of PNS in patients with TGCT by oncologists via testing for these antibodies may reduce long-term neurological dysfunction, a major cause of morbidity and mortality in our cohort, especially with the favorable TGCT outcomes.

The type of neurological manifestation and neural antibodies varied according to the TGCT subtype. Thus, the presence of neurological manifestations and neural antibodies may not only indicate an underlying occult TGCT but could also suggest the tumor histology. Potential study limitations include selection bias and referral bias. Although most patients achieved positive cancer outcomes, many faced long-term neurological disability.

## Notes

Supplement 1. eAppendix. Supplemental Materials and Methods

Supplement 2. Data Sharing Statement

## References

[1] Shah S, Flanagan EP, Paul P, . Population-based epidemiology study of paraneoplastic neurologic syndromes. Neurol Neuroimmunol Neuroinflamm. 2021;9(2):e1124. doi:10.1212/NXI.0000000000001124 34937736 PMC8696552

[2] Dubey D, Wilson MR, Clarkson B, . Expanded clinical phenotype, oncological associations, and immunopathologic insights of paraneoplastic Kelch-like protein-11 encephalitis. JAMA Neurol. 2020;77(11):1420-1429. doi:10.1001/jamaneurol.2020.2231 32744608 PMC7653501

[3] Voltz R, Gultekin SH, Rosenfeld MR, . A serologic marker of paraneoplastic limbic and brain-stem encephalitis in patients with testicular cancer. N Engl J Med. 1999;340(23):1788-1795. doi:10.1056/NEJM199906103402303 10362822

[4] Dubey D, Kryzer T, Guo Y, . Leucine zipper 4 autoantibody: a novel germ cell tumor and paraneoplastic biomarker. Ann Neurol. 2021;89(5):1001-1010. doi:10.1002/ana.26050 33583072

[5] Mandel-Brehm C, Dubey D, Kryzer TJ, . Kelch-like protein 11 antibodies in seminoma-associated paraneoplastic encephalitis. N Engl J Med. 2019;381(1):47-54. doi:10.1056/NEJMoa1816721 31269365 PMC6800027

