# Supplementary material for: Testicular Cancer–Associated Paraneoplastic Neurologic Syndromes
Source: JAMA Netw Open. 2025 Oct 21;8(10):e2538584. doi: 10.1001/jamanetworkopen.2025.38584 (PMC12541532; doi:10.1001/jamanetworkopen.2025.38584)
Supplement: Supplement 1. — eAppendix. Supplemental Materials and Methods [file jamanetwopen-e2538584-s001.pdf]

Supplemental Online Content

Harahsheh E, Hammami MB, Gupta P, et al. Testicular cancer–associated paraneoplastic neurologic syndromes. *JAMA Netw Open*. 2025;8(10):e2538584.
doi:10.1001/jamanetworkopen.2025.38584

eAppendix. Supplemental Materials and Methods

This supplemental material has been provided by the authors to give readers additional information about their work.

© 2025 Harahsheh E et al. *JAMA Network Open*.

Supplemental Materials and Methods

Study design and patient population

This retrospective cohort study included patients aged 18 and above with histopathologically confirmed TGCT or regressed TGCT, and PNS, regardless of neural antibody seropositivity in their sera and/or cerebrospinal fluid (CSF). Regressed TGCT is defined as a germ-cell tumor that has completely or partially regressed, without any intervention, leaving a scar in the parenchyma with or without vestiges of germ-cell tumor components. To be classified as a PNS, a case must meet the criteria outlined in the updated diagnostic framework published by Graus et al. in 2021.[6] Additionally, PNS-Care score, a score proposed by experts in the field of neuroimmunology that classifies PNS cases into three levels of diagnostic certainty (definite >=8, probable 6-7, possible 4-5) based on clinical phenotype, presence or absence of neuronal antibodies, and presence or absence of cancer, was calculated for each patient included in this study to assess the PNS diagnostic certainty for each case. Included patients were evaluated and treated at the Mayo Clinic (Rochester, Jacksonville, Phoenix) from January 1, 1990, to March 30, 2023.

Patients were identified via Mayo Data Explorer, a research tool available at the Mayo Clinic Healthcare System to identify and review patients' electronic medical records, using terms like paraneoplastic neurological syndromes, germ cell tumors, testicular tumors, seminoma, and non-seminomatous tumors, and through the Mayo Clinic Neuroimmunology laboratory database. For eligible TGCT and PNS patients, sera or CSF were tested for neural antibodies, including KLHL11-IgG, LUZP4-IgG, and Ma2-IgG. Additionally, given the recent discovery of KLHL11-IgG and LUZP4-IgG antibodies, archived sera of 36 patients with documented seronegative

© 2025 Harahsheh E et al. *JAMA Network Open*.

TGCT and PNS were tested for KLHL11-IgG, LUZP4-IgG, along with Ma2-IgG. Testing for neural antibodies involved the use of indirect immunofluorescence assay performed on a composite substrate of mouse cerebellum, midbrain, basal ganglia, thalamus, cerebral cortex, hippocampus, stomach, and kidney to detect IgG autoantibodies binding selectively to neuronal and glial antigens. Testing also included cell-based assays of HEK293-transfected cells for cell surface antibodies. For eligible patients, charts were reviewed independently by two authors (E.H, P.G.) for demographic factors (age at the time of TGCT diagnosis), oncological data (type of TGCT, pathologic stage [as defined by the American Joint Commission on Cancer (AJCC) seventh edition][9], risk stratification for TGCT [as defined by International Germ Cell Cancer Consensus Group Classification (IGCCCG)][10], surgical treatment, use of radiation or chemotherapy, relapse and mortality), and neurological data (neurological manifestations, presence of neurological symptoms either before or after TGCT diagnosis, duration from neurological symptoms to cancer diagnosis and vice versa, type of neural antibodies if present, treatment received for PNS and neurological outcome) whenever available. Outcomes of TGCT were categorized into four groups: remission, cure, stable or relapse. Cure was defined as no clinical, laboratory, or imaging evidence of tumor for at least 5 years following initial treatment, while remission was defined as the absence of clinical, laboratory, or imaging evidence of tumor for less than five years following initial treatment. Patients with only residual laboratory evidence of tumor following initial treatment for less than five years (i.e. elevated alpha fetoprotein (AFP) or beta-human chorionic gonadotropin (b-HCG)) undergoing routine serological and radiological surveillance, were considered stable. Neurological outcomes were classified as improvement, progression, or disease stability based on the assessment of the treating neurologists at our institution following initiation of immunomodulatory treatment

© 2025 Harahsheh E et al. *JAMA Network Open*.

compared to the patients' baseline neurological status at the time of PNS diagnosis or initial encounter.

Statistical analysis

Demographic and baseline clinical characteristics for the entire cohort were described and summarized using frequencies, percentages, means, standard deviations (SD), medians, or interquartile ranges (IQR) as deemed appropriate. For time analyses between TGCT diagnosis and neurological symptom onset, time zero was set as the time of onset of TGCT diagnosis. Pearson's Chi-square test and Fischer's exact test were used to identify statistical differences between categorical variables. A p-value threshold of ≤ 0.05 was considered indicative of statistical significance. Results reporting are- adherent to STROBE guidelines.

Ethics statement

The institutional review board at Mayo Clinic reviewed and approved the study (IRB Protocol number 21-010106). All Mayo Clinic patients gave written consent for this research.

© 2025 Harahsheh E et al. *JAMA Network Open*.
