# Supplementary material for: Testicular Cancer–Associated Paraneoplastic Neurologic Syndromes
Source: JAMA Netw Open. 2025 Oct 21;8(10):e2538584. doi: 10.1001/jamanetworkopen.2025.38584 (PMC12541532; doi:10.1001/jamanetworkopen.2025.38584)
Supplement: Supplement 2. — Data Sharing Statement [file jamanetwopen-e2538584-s002.pdf]

Data Sharing Statement

Harahsheh. Testicular Cancer–Associated Paraneoplastic Neurologic Syndromes. *JAMA Netw Open*. Published October 21, 2025. doi:10.1001/jamanetworkopen.2025.38584

Data
Data available: Yes
Data types: Deidentified participant data
How to access data: Dubey.Divyanshu@mayo.edu
When available: With publication

Supporting Documents
Document types: None

Additional Information
Who can access the data: researchers whose proposed use of the data has been approved
Types of analyses: For a specified purpose
Mechanisms of data availability: Signed data access agreement
